# Post-processed data and graphical tools for a CONUS-wide eddy flux evapotranspiration dataset

**DOI:** 10.1016/j.dib.2023.109274

**Published:** 2023-05-30

**Authors:** J.M. Volk, J.L. Huntington, F. Melton, B. Minor, T. Wang, S. Anapalli, R.G. Anderson, S. Evett, A. French, R. Jasoni, N. Bambach, W.P. Kustas, J. Alfieri, J. Prueger, L. Hipps, L. McKee, S.J. Castro, M.M. Alsina, A.J. McElrone, M. Reba, B. Runkle, M. Saber, C. Sanchez, E. Tajfar, R. Allen, M. Anderson

**Affiliations:** a Desert Research Institute, 2215 Raggio Pkwy, Reno, NV 89512 USA; b NASA Ames Research Center, Mail Stop 245-1, Moffett Field, CA 94035-1000 USA; c California State University, Monterey Bay, Seaside, CA 93955 USA; d University of California, Berkeley, Berkeley, CA 94720 USA; e USDA-ARS, Sustainable Water Management Research Unit, 4006 Old Leland Road, Stoneville, MS 38776 USA; f USDA-ARS US Salinity Laboratory, Agricultural Water Efficiency and Salinity Research Unit, 450 W Big Springs Rd Riverside, CA 92507-4617 USA; g USDA-ARS Conservation & Production Research Laboratory, 300 Simmons Road, Bushland, TX 79012 USA; h USDA-ARS US Arid-Land Agricultural Research Center, 21881 North Cardon Lane, Maricopa, AZ, 85238 USA; i University of California, Davis, CA 95616 USA; j USDA-ARS Hydrology and Remote Sensing Laboratory, Bldg. 007, Rm. 104, BARC-West, Beltsville, MD 20705-2350 USA; k USDA-ARS National Laboratory for Agriculture and The Environment, 1015 N. University Blvd., AMES, IA 50011 USA; l Utah State University, Logan, UT 84322-0102 USA; m E & J Gallo Winery, Viticulture, Chemistry and Enology, Modesto, CA USA; n USDA-ARS Crops Pathology and Genetics Research Unit, Davis, CA, 95616 USA; o USDA-ARS Delta Water Management Research, Jonesboro, AR, 72401 USA; p University of Arkansas, Fayetteville, AR 72701 USA; q University of Arizona, Tucson, AZ 85721 USA; r University of Idaho, Moscow, ID 83844 USA; s University of Nebraska-Lincoln, Lincoln, NE 68588 USA

**Keywords:** Flux data, Energy balance closure, Meteorological, Evapotranspiration (ET), Post-processing

## Abstract

Large sample datasets of *in situ* evapotranspiration (ET) measurements with well documented data provenance and quality assurance are critical for water management and many fields of earth science research. We present a post-processed ET oriented dataset at daily and monthly timesteps, from 161 stations, including 148 eddy covariance flux towers, that were chosen based on their data quality from nearly 350 stations across the contiguous United States. In addition to ET, the data includes energy and heat fluxes, meteorological measurements, and reference ET downloaded from gridMET for each flux station. Data processing techniques were conducted in a reproducible manner using open-source software. Most data initially came from the public AmeriFlux network, however, several different networks (e.g., the USDA-Agricultural Research Service) and university partners provided data that was not yet public. Initial half-hourly energy balance data were gap-filled and aggregated to daily frequency, and turbulent fluxes were corrected for energy balance closure error using the FLUXNET2015/ONEFlux energy balance ratio approach. Metadata, diagnostics of energy balance, and interactive graphs of time series data are included for each station. Although the dataset was developed primarily to benchmark satellite-based remote sensing ET models of the OpenET initiative, there are many other potential uses, such as validation for a range of regional hydrologic and atmospheric models.

**Specifications Table**

**Table:** 

Subject	Earth and Planetary Science
Specific subject area	Atmospheric Science
Type of data	Tabular time series data, table, and interactive graphics
How the data were acquired	Open-path eddy covariance systems, Bowen-ratio technique, and weighing lysimeter methods
Data format	Secondary data in CSV, XLSX, and HTML format
Description of data collection	Most primary data was downloaded directly from the public AmeriFlux network, which hosts eddy covariance system data collected across a range of land cover types. Additional primary data was provided directly from principal investigators who oversee eddy covariance and other instrumentation measuring in situ evapotranspiration. Data collection was limited to stations located within the contiguous USA. Gridded meteorological data were downloaded from the THREDDS Data Server hosted by Northwest Knowledge Network at the University of Idaho (https://thredds.northwestknowledge.net/).
Data source location	*Primary data sources*: • *AmeriFlux network* (https://ameriflux.lbl.gov/) • *California State University, Monterey Bay, Seaside, CA, USA* • *Desert Research Institute, Reno, NV, USA* • *gridMET, Northwest Knowledge Network at the University of Idaho* (https://thredds.northwestknowledge.net/)
	• *United States Geological Survey Nevada Water Science Center, Carson City, NV, USA* • *Delta-Flux network, Arkansas, Louisiana, MS, USA* • *United States Department of Agriculture Agricultural Research Service (USDA-ARS):* • *Sustainable Water Management Research Unit, Stoneville, MS, USA* • *US Salinity Laboratory, Agricultural Water Efficiency and Salinity Research Unit, Riverside, CA, USA* • *Conservation & Production Research Laboratory, Bushland, TX, USA* • *US Arid-Land Agricultural Research Center, Maricopa, AZ, USA* • *Hydrology and Remote Sensing Laboratory, Beltsville, MD, USA*
Data accessibility	Repository name: Zenodo Data identification number: 10.5281/zenodo.7636781 Direct URL to data: http://zenodo.org/record/7636781
Related research article	Volk, J. M., Huntington, J., Melton, F. S., Allen, R., Anderson, M. C., Fisher, J. B., ... & Kustas, W. (2023). Development of a Benchmark Eddy Flux Evapotranspiration Dataset for Evaluation of Satellite-Driven Evapotranspiration Models Over the CONUS. Agricultural and Forest Meteorology (331), http://doi.org/10.1016/j.agrformet.2023.109307

## Value of the Data

• Accurate *in situ* estimates of evapotranspiration (ET) are critical for atmospheric and hydrologic research and operational applications.
• Data includes ET, surface energy and heat fluxes, and meteorological data from 161 stations (148 eddy covariance).
• Data post-processing was conducted using open-source software and subject to manual quality control checks.
• ET (latent heat flux) was corrected for energy balance closure error; closure metrics for each flux site are included.
• The data is useful for benchmarking atmospheric and hydrologic models.
• Site diagnostics and meteorological data can be used for research applications such as energy balance closure analysis.

## Objective

The production of this dataset was a foundational step in a large-scale effort to develop data and tools to be used in the evaluation of remotely sensed ET estimated from the models of the OpenET initiative [1,2]. As part of that goal, we also seek to make the data well curated, documented, and accessible to the wider community. Volk et al. [1] describes the entire process and rationale of decisions in making this data and includes analysis of energy balance closure error and flux-footprint prediction methods. This article compliments Volk et al. [1] by going into more depth on the technical details of eddy flux data post-processing specifically, including the calculation of ancillary meteorological data and equations. This article also provides user-oriented descriptions of data and graphics production, accessibility, and file formats.

## 1 Data Description

**Fig. 1:**
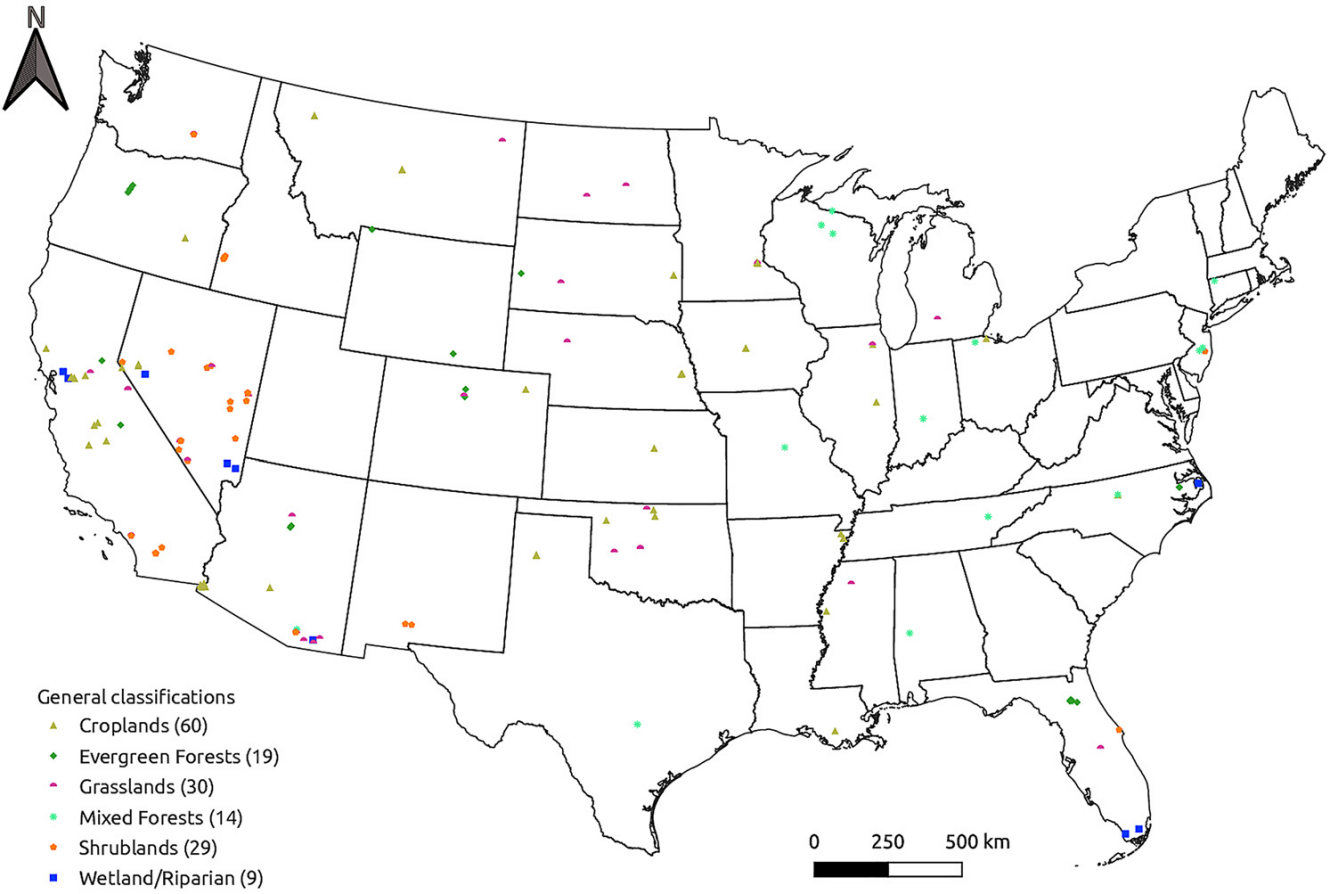
Map showing the distribution of ET stations included in the dataset and their general land classification, including the total number of stations in each classification (in legend).

This data primarily consists of post-processed, daily and monthly aggregated, measurements of evapotranspiration (ET), latent, sensible, and soil heat fluxes along with net radiation measurements from 161 stations (primarily AmeriFlux eddy covariance towers) distributed across the contiguous United States (Fig. 1). It also includes meteorological measurements, derived atmospheric variables, and interactive graphics of such data for each station.

A summary metadata table is included with the dataset to provide site information for all stations. Table 1 shows a subset of the metadata, which includes information for each site (per row), including an identifier, data provider and contact, land cover, data start and end dates, and energy balance closure results.

**Table 1:** A subset of information as found in the station metadata file that is included with the dataset. Additional columns that are in the file but are not shown below include information about data providers including principal investigator, contact email, and DOIs.

Site ID	General classification	State	Data source/network	Period of record	Energy balance	Latitude	Longitude	Elevation (m)	Land cover details	Land cover type	Measurement technique
US-A32	Grasslands	OK	AmeriFlux	06/2015-06/2017	0.90	36.819268	-97.819772	335	Hay pasture	Grasslands	Eddy covariance
US-A74	Croplands	OK	AmeriFlux	01/2016-10/2017	0.92	36.808464	-97.548854	337	Sorghum	Annual crops	Eddy covariance

Daily and monthly fluxes and meteorological data were written to CSV files and included in interactive graphics using a common naming scheme. A key to the standard naming scheme used in data files is provided with the data. The full list of daily and monthly meteorological data that are included for each station (depending on the initial data availability) are the following: latent heat flux (LE), sensible heat flux (H), net radiation (R_n_), and soil heat flux (G); shortwave and longwave radiation; potential solar radiation; air temperature (average, minimum, maximum, and dew point); wind speed; station and gridMET [3] precipitation; vapor pressure and vapor pressure deficit; LE and H after correction for energy balance closure; soil moisture; energy balance ratio; ET; fraction of reference ET; ET before and after energy balance correction; station-derived and gridMET reference ET; and ET gap-filling information.

In addition to the station metadata, a table that lists and explains atmospheric variables used throughout the dataset is included with the data. This file lists all standard variable names (e.g., “ppt” for precipitation) which are found in CSV time series files and HTML graphics. The table also includes a short description of each variable and their units (which were converted, if necessary, to ensure consistent variable units across all stations).

**Fig. 2:**
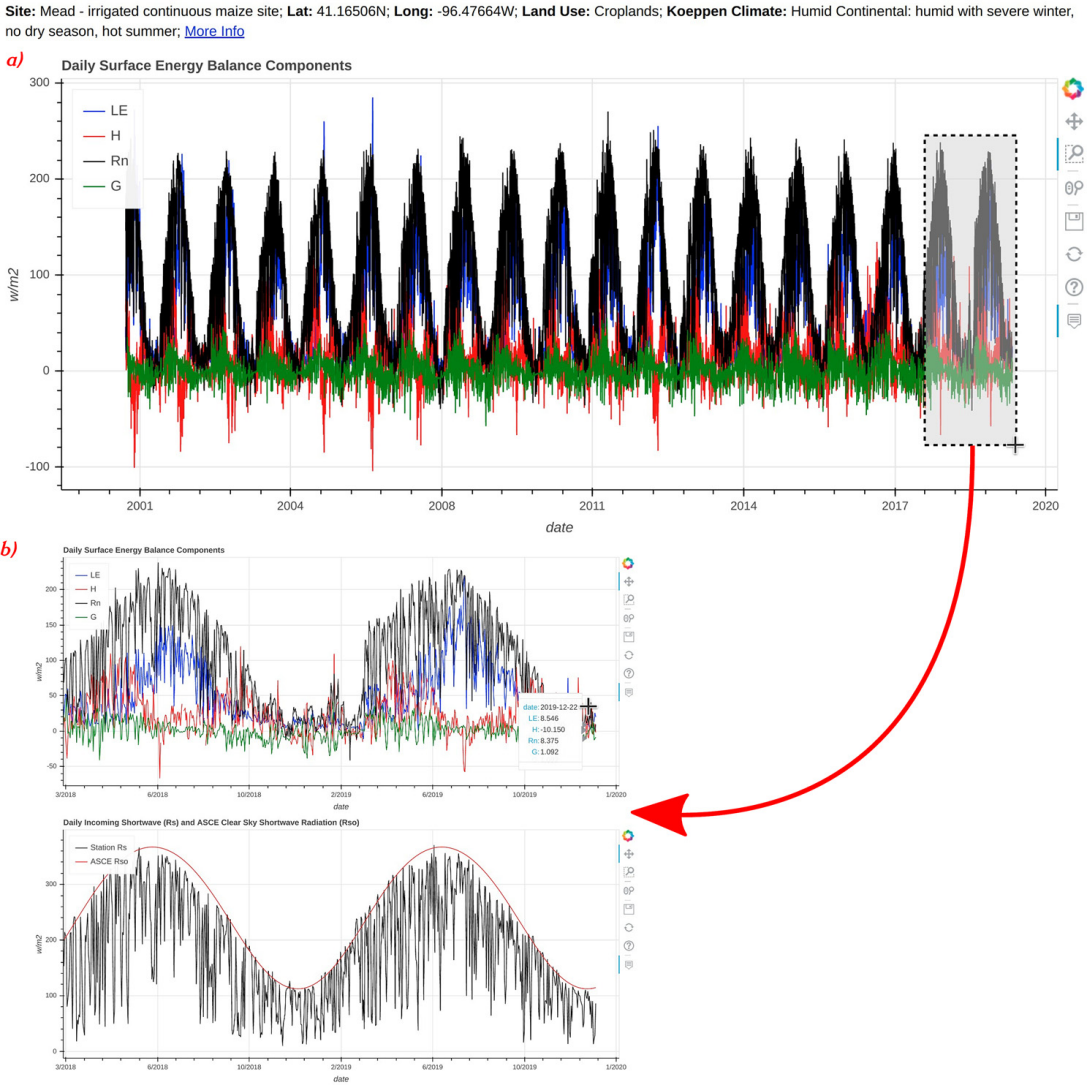
A screenshot of select subplots included in the HTML graphics for AmeriFlux site “US-NE1”. Subplot (a) (top) shows the daily surface energy balance; and (b) (bottom) shows the zoomed in area from plot (a) with subsequent plots automatically zoomed into the same temporal period. Plot (a) includes site metadata in the header as well as a link titled “More Info,” which directs to the AmeriFlux website for the station, plot (b) also shows the cursor hover information which displays the date and values for all data on the plot where the cursor is held.

Interactive graphical diagnostics were made for each station and are in the form of HTML files, which can be viewed with a web browser. Features of HTML graphics include panning and zooming with time axes paired among all daily or monthly time series plots. For example, if one zooms into a subset of ET data over a specific period, all other monthly time series plots will be adjusted simultaneously to the same time window (Fig. 2).

The legends to all subplots within the HTML graphics files are also interactive such that they can be modified to show a subset of variables by double-clicking on legend items. Important station metadata such as land cover, latitude, and longitude coordinates, and for most AmeriFlux stations, the Köppen Climate, and a link to the site-specific AmeriFlux website are all displayed near the top of each HTML graphic file (Fig. 2).

Data coverage varies by station; however, all atmospheric measurements fall within the period of 1995-2021. This includes a total of 212,273 days of average ET values that were not gap-filled. Specifically, there are 60 stations classified as croplands with a combined total of 65,631 days of ET; 19 evergreen forests (36,147 days); 30 grasslands (37,708); 14 mixed forests (24,733); 29 shrublands (35,510); and 9 wetland/riparian (12,544) (Fig. 1).

## 2 Experimental Design, Materials and Methods

### 2.1 Data collection and background

Data collected from eddy covariance (EC) systems equipped with a 3-dimensional sonic anemometer, infrared gas analyzer, net radiometer, and at least one soil heat flux plate were collated from multiple providers (listed in specifications Table) [4]. Data for most sites (106 of 161 sites) were initially directly downloaded from the AmeriFlux network website, accessed on October 27^th^, 2020 [5]. Acknowledgements for all AmeriFlux stations are included below; station principal investigator (PI) name and contact as well as AmeriFlux DOIs are also included in the metadata file that is part of the dataset. In addition to EC data, 8 Bowen ratio instrumented stations in Nevada operated by the United States Geological Survey and 4 precision weighing lysimeters stations in Texas [6] are included in the dataset. Instrumentation at the Bowen Ratio sites included two solid-state temperature and relative-humidity sensors mounted at two heights typically with 3.3 ft spacing between them, with the lower sensor set at 1.6 ft [7] or 3.3 ft [8] above the vegetation canopy. The sensors were installed on top of a mechanism that interchanged the sensor positions halfway through each measurement period so that biases could be minimized, and accurate temperature and vapor pressure gradient data could be obtained to compute the Bowen Ratio. The Bowen Ratio stations were also equipped with a net radiometer, a pair of thermocouples, soil-heat flux plates, a water content reflectometer, and either a 3-cup rotor or marine-grade propeller type anemometer. Lysimeters were instrumented with soil heat flux plates, time-domain reflectometry (TDR) soil water sensors, albedometer, pyrgeometer, relative humidity and air temperature sensors, wind velocity sensor, and infrared thermometers (one aimed obliquely and one aimed nadir). Lysimeter surface area was approximately 9 m^2^, the undisturbed soil monolith in each lysimeter was 2.3-m deep, and lysimeters were under constant vacuum drainage. Lysimeters were tilled, fertilized, planted, and treated with pesticides comparably to the surrounding fields. More background on the Bowen Ratio and lysimeter stations and data are in the supplementary materials Text S1 and S2 in Volk et al. [1]. Additional background information on non-AmeriFlux EC systems, including for cropland sites in southwestern Arizona, GRAPEX vineyard sites in California [9], and sites in central Oregon are in Texts S3, S4, and S5 in Volk et al. [1]. Background on EC sites from the Delta-Flux network, which covers sites within the Mississippi River Alluvial Plain, can be found in Runkle et al. [10]. Data not downloaded directly from the AmeriFlux network were directly provided by site PI's and network administrators. Almost all data collected were at half-hourly temporal frequency, a typical averaging period for EC high-frequency data processing software, such as EddyPro (LI-COR) [5]. The exceptions to this were a few EC sites at hourly frequency and some Bowen ratio stations at a daily frequency.

Initial data processing steps and data quality assessments were performed for the Bowen Ratio and lysimeter station data by their station teams prior to gap-filling and visual inspection described below in Sections 2.2.2 and 2.2.7.

At the Bowen Ratio stations, wind speed was used to correct net radiation following the steps outlined in the Campbell Scientific NR-Lite net radiometer manual. Readings from the instruments were recorded every 10 seconds and averaged over a 20-minute period to obtain energy fluxes. Raw 20-minute energy flux and ET values were compiled for Bower Ratio sites ET_1 and ET_8 [11] and B_01, B_11, and TAM [8]. Raw daily ET values were compiled at sites MOVAL, MR, and VR [7] because the sub-hourly data was not readily available.

The weighing lysimeters were calibrated using masses traceable to NIST, and calibration accuracy was 0.04 mm or better. The ET data were averaged to daily (midnight to midnight) values from 15-minute measurements. Eight neutron probe access tubes in each field around a lysimeter were used to determine ET by soil water balance about weekly to verify representativity of the accumulative lysimeter ET data [12].

### 2.2 Data processing

Post-processing of EC data, including gap-filling, temporal aggregation, energy balance closure corrections, data filtering, and calculation of atmospheric variables, was conducted in a reproducible way using the Python 3 [13] open-source software “flux-data-qaqc” version 0.1.6 [14]. The software is hosted on GitHub and the Python Package index. It also has thorough online documentation with a user tutorial.

#### 2.2.1 Data selection

Before post-processing of flux and atmospheric variables, data availability from each station was identified. Our main requirement was that each EC station has overlapping records (at least partially) of the four main surface energy balance components: LE, H, R_n_, and G. In addition to energy balance fluxes, soil heat storage and on-tower meteorological measurements of air temperature, relative humidity, vapor pressure, vapor pressure deficit, short and longwave radiation (incoming and outgoing), precipitation, wind speed, wind direction, and soil moisture were also ingested when available.

A procedure to automatically select or average the appropriate input variables was used to parse AmeriFlux data when more than one sensor recording exists at a station (for the same variable). Data providers sometimes report measurements from multiple sensors measuring the same environmental variable. The data selection algorithm we developed involves a chain of preferences and generally favors site PI-approved, aggregated, and gap-filled measurements. We took the average of all records if no PI-approved record for a variable was given and multiple sensors were used, which was most common for soil heat flux measurements. The result is a single estimate of the four energy balance components for each time step used in the energy balance closure correction and ET calculation. The algorithm for selecting variables is as follows:

1. If a *single* PI provided variable exists, choose it
2. If multiple PI provided variables exist, account all PI gap-filled and spatially aggregated versions, then follow the rules below:
  (a) If PI gap-filled and spatially aggregated variables exist take the average (or single time series if only one) of them
  (b) Else if only PI gap-filled exist use or take the average if multiple
  (c) Else if only PI spatially aggregated variables exist use or take the average
  (d) Otherwise use or take the average of any other PI-provided versions
3. If no PI-provided versions exist follow similar substeps as step #2 based on the qualifiers, in this case:
  (a) If gap-filled and spatially aggregated variables exist take the average (or single time series if only one) of them
  (b) Else if only gap-filled exist use or take the average if multiple
  (c) Else if only spatially aggregated variables exist use or take the average
  (d) Otherwise take the average of any versions

For EC data acquired outside the AmeriFlux network, we used flux data as provided and suggested directly by the site's PI and team. If corrected data were provided, e.g., using Webb, Pearman and Leuning density corrections [15], they were chosen over non-corrected fluxes.

#### 2.2.1 Meteorological variable calculations

Vapor pressure, vapor pressure deficit, saturation vapor pressure, air temperature, dew point temperature, relative humidity, and potential solar radiation were estimated using well-established methods, including those set by the American Society of Civil Engineers (ASCE) in the standardized reference ET equation report [16]. We note that estimated meteorological variables are ancillary to the primary post-processed ET dataset, and the estimation methods employed may not be the best suited for certain applications. These variables were estimated primarily to serve as diagnostics for visually assessing energy balance and ET data quality.

Minimum and maximum air temperature were rarely provided with raw data (e.g., at half-hourly timesteps); however, their daily values were estimated as the minimum and maximum values every 24 hours. Half hourly or hourly saturation vapor pressure was computed following the Tetens approximation, which is accurate for most surface air temperatures,

(1) $$es\;=\;0.6108e^{\left(\frac{17.27T}{T\;+\;237.3}\right),}$$

where, es is saturation vapor pressure [kPa] and T is air temperature [C]. The definition of relative humidity was sometimes used to estimate actual vapor pressure or relative humidity depending on which data are available,

(2) $$rh\;=\;\frac{e}{es},$$

where, rh is relative humidity as a fraction [-], and e is actual vapor pressure [kPa]. The Clausius-Clapeyron relation was used to estimate dew point temperature at half-hourly or hourly timesteps,

(3) $$T_{dew}\;=\;{\left[\frac{1}{T_{0}}\;-\frac{R_{v}}{L_{v}}\cdot ln\left(\frac{e}{e_{0}}\right)\right]}^{-1},$$

where, $T_{dew}$ is dew point temperature [K], $T_{0}$ is 273.15 [K], $R_{v}$ is the universal gas constant of water vapor 461 [J K^−1^ kg^−1^], $L_{v}$ is the specific latent heat of vaporization of water 2.5×10^6^ [J kg^−1^], and $e_{0}$ is 0.6113 [kPa].

Potential or clear sky solar radiation and Penman-Monteith standardized reference ET were estimated using methods from Allen et al. [16]. These methods were translated to Python by the “refet” library (https://github.com/WSWUP/RefET), which was used within the “flux-data-qaqc” package for generating this dataset. Daily clear sky radiation was estimated as

(4) $$R_{so}\;=\;\left(0.75\;\times \;2\times 10^{-5}z\right)R_{a},$$

where, $R_{so}$ is clear sky radiation [MJ m^−2^ d^−1^], z is elevation above sea level [m], and $R_{a}$ is extraterrestrial radiation [MJ m^−2^ d^−1^]. Here, $R_{a}$ is calculated using a simple approximation that is a function of latitude, day of the year, and time of day, per Eqs. 21-29 in Allen et al. [16], which are not listed here for brevity.

For reference purposes only, daily ASCE standardized Penman-Monteith short (grass) reference ET (ETo) [mm d^−1^] was calculated for sites with sufficient input data. See Eq. 1 in Allen et al. [16] for the full formula and detailed explanations. Daily inputs are minimum and maximum air temperature, incoming shortwave radiation, actual vapor pressure, and average horizontal wind speed. Other inputs are computed from the day of the year, time of day, site elevation, and latitude. The height of the 3D sonic anemometer (if known) was used to adjust wind speed to a height of 2 meters, assuming a logarithmic vertical velocity profile (Eq. 33 in Allen et al. [16]). Saturation vapor pressure for ASCE ETo was calculated as the average vapor pressure using both daily minimum and maximum air temperatures as input in Eq. 1. Please note that many sites do not fit the requirements for the ASCE ETo equation (e.g., being well watered short grass). Also, the daily ETo formulation may introduce bias and uncertainty particularly in the winter when daylight hours are lessened resulting in skewed daily average radiation and temperature. We applied the daily ETo formulation using daily averaged inputs to be consistent with other meteorological calculations, however, future dataset versions may include improvements to this approach.

#### 2.2.2 Gap-filling, daily averaging, conversions, and renaming of initial data

Initial half-hourly or hourly energy balance variables, as well as inputs for the ASCE standardized reference ET equation, were gap-filled. Specifically, the variables LE, H, R_n_, G, minimum, maximum, and average air temperature (min and max used to estimate daily vapor pressure), incoming shortwave radiation, and wind speed were gap-filled. For EC stations that recorded soil heat storage above soil heat flux plates, G was adjusted (heat storage values were added to G) to account for storage before gap-filling. We used simple linear interpolation for gap-filling initial energy balance and reference ET data and set the maximum length of gaps that would be filled overnight versus daytime periods. The daytime was defined as the periods where R_n_≥0 and nighttime when R_n_ <0, and the nighttime gap-filling window was based on 12:00 PM–12:00 PM daily intervals or noon to noon whereas the daytime window was from midnight to midnight. Gaps of up to 4 hours were filled during nighttime periods and 2 hours during daytime, respectively, and the total number of sub-day gaps were also computed per day and saved. After the gap-filling procedure, if any date had remaining gaps, e.g., a 3-hour daytime gap in LE flux, the values for that date were removed before the daily aggregation. In other words, the half-hourly values for such dates were all set to null before computing 24-hr aggregates to avoid skewing daily estimates on dates with many gaps. Daily time series were then computed as 24-hr averages or totals for all input variables, for example, energy balance components were computed as averages, whereas precipitation was summed. Data filtering based on sub-daily gaps was only performed for the variables mentioned above, namely, energy balance components and some reference ET input variables. Other ancillary meteorological variables such as vapor pressure deficit were averaged or summed over calendar days regardless of their number of half-hourly or hourly gaps.

Data that was initially collected from data providers were checked for units and sometimes converted using automated methods of the “flux-data-qaqc” software. A key that explains all calculated meteorological variables is included with the dataset and can also be found in the online documentation. Input data precision was kept throughout all processing and computational steps, the determination of appropriate significant figures for different applications is best chosen by the user for each specific data application.

Ingested and computed variables were subject to a standardized naming scheme and strict unit assignments. A data legend (provided) lists names, units, and brief descriptions of all flux, meteorological, ET, and QA/QC related variables that are part of the dataset. A given station may not include all of these variables due to data availability and site instrumentation. Sometimes additional data may appear in data files when multiple records were used to average the same atmospheric variable. For example, if multiple air temperature measurements were included at different heights or locations and their average was selected to use for meteorological variable calculations, then all individual records would be included in the post-processed data using their initial names (e.g., “T_1_1_1”, “T_1_2_1”, … following AmeriFlux naming standards) and the average result would be renamed as “t_avg” following the standardized naming scheme.

#### 2.2.3 Gridded climate data

Daily gridMET [3] precipitation [mm] and grass and alfalfa ASCE reference ET (ETo and ETr) [mm d^−1^] data were downloaded for all sites over their respective period of record. These data were downloaded directly from the THREDDS Data Server hosted by Northwest Knowledge Network at the University of Idaho (https://thredds.northwestknowledge.net/). Daily time series of gridMET variables were selected for each station by querying data from the gridMET pixel whose centroid coordinates are nearest to the stations’ coordinates. gridMET resolution is 1/24 decimal degrees or approximately 4 km. For daily ET gap-filling purposes and as an effort to develop a complete dataset, we downloaded gridMET ETo for each location even though most do not satisfy the site requirements of well-watered short grass.

#### 2.2.4 Energy balance closure assessment and correction

Daily average energy balance data were used to correct daily average turbulent fluxes (LE and H) for energy balance closure error. The technique used is based on the energy balance ratio approach used to process daily data for the FLUXNET2015 dataset and the ONEFlux data processing pipeline (https://fluxnet.org/data/fluxnet2015-dataset/data-processing/) with slight modifications [5].

The calculation begins with the computation of the daily energy balance ratio (EBR) [-]

(5) $$EBR\;=\;\frac{LE\;+\;H}{R_{n}\;-\;G}.$$

The result of the closure correction procedure is daily correction factors for LE and H that are based on the reciprocal of the daily EBR. When these correction factors are applied to the initial LE and H, the energy balance closure is improved. Because the EBR values used in the correction are not the original EBR values but rather a filtered and gap-filled version based on sliding windows (as described below), the final energy balance closure is not always perfect but averages to near perfect over the sliding window time periods (about 15 days). The “flux-data-qaqc” Python package has online documentation that includes a visual description of the closure correction steps below.

First, the daily EBR outlier values were removed using a threshold of 1.5 times the interquartile range,

(6) $$Q_{1}-1.5\cdot IQR\;\leq \;EBR\;\leq \;Q_{3}+1.5\cdot IQR,$$

where $Q_{1}$ is the first quartile of EBR values, $Q_{3}$ is the third quartile, and IQR is the interquartile range or $Q_{3}-Q_{1}$.

After removing outlier EBR values, a series of sliding windows are used to make a smoothed time series of EBR. First the daily EBR gap count within a centered 15 day sliding window is counted. Gaps in the daily EBR time series may exist due to gaps in one or more of the four main energy balance variables. If the gap count is less than 4, i.e., 11 or more valid EBR values exist, the median value is calculated. Median EBR values from the 15-day sliding window are also checked and filtered out if they met any of the following criteria:

(7) $$\left|\frac{1}{EBR}\right|\;\geq \;2,$$

(8) $$\left|\frac{1}{EBR}\right|\;\leq \;0.5,$$

(9) $$\left|\frac{1}{EBR}\right|\times LE\;\geq \;800\;\left[W\;m^{-2}\right],$$

(10) $$\left|\frac{1}{EBR}\right|\;\times LE\;<\;-100\;\left[W\;m^{-2}\right],$$

where the LE values are paired with the corresponding EBR values, i.e., they occur on the same date. If the median values were removed based on these criteria or less than 11 valid EBR values are in the window, the average EBR value is taken from a centered 11-day sliding window. The same outlier criteria listed in Eqs. 7-10 are applied to the 11-day average EBR values. If there are no valid EBR values within the smaller 11-day window, or if the average fails the outlier criteria, then the last option using the climatology of EBR is used. The EBR climatology is calculated by first taking the average for each day of the year using values from all years on record; the values used in the day of year average have already been filtered following the steps above. Then the average from a centered 11-day sliding window from the day of year average (climatology) is used to calculate an EBR value. This step will fill any remaining daily EBR gaps unless there are no valid values for a specified day of the year. At this stage in the energy balance closure correction, we have a filtered, smoothed, and gap-filled daily time series of EBR values which is denoted as $EBR_{corr}$. These values are used as correction factors for turbulent fluxes

(11) $$ebc_{cf}\;=\;\frac{1}{EBR_{corr}},$$

where $ebc_{cf}$ is the energy balance closure correction factor time series. The correction factors are applied to initial turbulent fluxes

(12) $$LE_{corr}\;=\;LE\;\times \;ebc_{cf}$$

and

(13) $$H_{corr}\;=\;H\;\times \;ebc_{cf},$$

where LE and H are the original time series of daily average fluxes and $LE_{corr}$ and $H_{corr}$ are the time series that have been corrected for energy balance closure error.

#### 2.2.5 Calculations of ET and EToF

Initial and corrected daily average latent energy fluxes were used to calculate ET rates

(14) $$ET\;=\;\frac{LE}{\lambda }\times 86400$$

and

(15) $$ET_{corr}\;=\;\frac{LE_{corr}}{\lambda }\times 86400,$$

where ET and $ET_{corr}$ are initial and closure corrected evapotranspiration [mm d^−1^], and λ is the latent heat of vaporization [W s kg^−1^]. Air temperature was used to adjust λ following Harrison [17]

(16) $$\lambda \;=2501\times 10^{3}\;-\left(2361\times T_{avg}\right),$$

where $T_{avg}$ is the daily average air temperature [C]. On dates with missing air temperature measurements, 20 degrees celsius was used.

Daily fraction of reference ET (EToF) [-] was calculated using gridMET short reference ET (ETo) [mm d^−1^], and ET that was corrected for energy balance closure error [mm d^−1^]

(17) $$EToF\;=\;\frac{ET_{corr}}{ET_{o}}.$$

Station-based ETo was not used because not all EC stations had sufficient measurements to compute a complete time series of ETo; a complete time series without gaps was required because the EToF was subsequently used for daily gap-filling of $ET_{corr}$ (see Section 2.2.6). The daily time series of EToF underwent filtering and gap-filling, starting with the removal of outliers outside of 1.5 times the interquartile range using the same method as used for the initial EBR (Eq. 6). Next, the 7-day moving average was calculated using a centered window and requiring a minimum of 2 values in the window. The remaining gaps in the moving average were linearly interpolated and extrapolated.

#### 2.2.6 Daily ET gap-filling and monthly data aggregation

In addition to the initial gap-filling of LE and other variables, daily closure corrected ET estimates were gap-filled. A complete time series of daily ET estimates were computed as

(18) $$ET_{fill}\;=\;EToF_{filtered}\;\times \;ET_{o},$$

where $ET_{fill}$ is a daily ET time series which may be used for gap-filling $ET_{corr}$, $EToF_{filtered}$ is the filtered and gap-filled time series of EToF described in Section 2.2.5, and $ET_{o}$ is the downloaded time series of gridMET short reference ET (see Section 2.2.3).

Daily ET and other meteorological variables were aggregated to form monthly time series. Variables such as heat and energy fluxes, temperature, and other rates were averaged from daily time series, whereas magnitudes like precipitation and ET were summed over monthly periods. For all variables other than $ET_{corr}$, which was gap-filled using Eq. 18, a simpler gap-filling method was used before monthly aggregation. This method was as follows: 1) for each month, the number of missing days is counted; 2) if the number of daily gaps exceeds 20% of that month's total days, then the monthly aggregate is not computed and left as a gap in the monthly time series; 3) otherwise, the month's average daily value is computed and used to gap-fill all daily gaps before monthly aggregation.

#### 2.2.7 Visually based data filtering, site selection, and classification

Post-processed daily and monthly time series data were visually inspected using the interactive plots of meteorological and flux data. In particular, the energy balance closure corrected ET data was inspected for issues that may not have been remedied from the automated methods. Rarely, clear issues with data quality were identified and specific dates or periods of variables were subject to manual removal. For example, extreme data spikes, flat lines, or trends in individual energy balance components were sometimes removed on certain dates and the post-processing routine would be reiterated. For the Bowen Ratio and Lysimeter stations visual QA/QC was the only filter on erroneous data, however, the gap-filling and time aggregation steps outlined above were performed for these sites’ data.

Only EC sites that passed strict energy balance closure criteria were included in this dataset. Specifically, we computed average growing season and cold season energy balance closure from daily fluxes and required sites to have closure greater than 75% during the growing season and greater than 60% during the cold season. Growing season start and end dates were determined for each station using a cumulative growing degree day approach as described in Volk et al. [1]. Average closure results were estimated using the linear least-squares regression slope, forced through the origin (intercept = 0), of daily average available energy (Rn – G) against turbulent fluxes (LE + H). This method for estimating energy balance closure was computed using daily energy balance variables for each EC station; scatter plots of daily and monthly available energy versus turbulent fluxes and the linear regression line are also included in interactive graphics for each EC station.

Energy balance closure was the major criteria for removing stations from an initial pool of 328 EC systems [1]. Other instrumented stations, e.g., sites that use a residual energy balance approach or the Bowen Ratio technique to estimate LE, were filtered primarily from visual inspection of data and qualitative assessment. Many EC stations (≈ 22%) were missing measurements of one or more energy balance component (most commonly soil heat flux); therefore, energy balance closure could not be assessed, and they were not included in the final dataset. Approximately 32% of the initial EC stations were excluded because they did not meet the closure criteria, and 33 (or ≈ 10%) of the initial stations were held out of the dataset for a future blind model evaluation of OpenET remote sensing data [1,2]. About 16% or 24 additional EC stations were removed from the initial pool for other reasons including: insufficient data coverage due to excessive data gaps in the half-hourly records; visual/qualitative inspection of the data; and inappropriateness of site location for the EC technique. For example, sites located near steep transitions in topography such as within a canyon or valley or adjacent to large structures such as buildings that may affect the site's turbulence. Furthermore, a few stations were not included simply due to data sharing policies which may change in future versions.

Each ET station included in the dataset was inspected to identify its general and specific land cover and land use during its period of record. For AmeriFlux stations, this was conducted by investigating the metadata provided by the network (https://ameriflux.lbl.gov/data/badm/) as well as from visual inspection of site images, google earth and other satellite imagery, contacting site PIs, and literature review. For non-AmeriFlux sites, land cover and land use information were provided by data providers. Station general classifications were croplands, grasslands, shrublands, mixed forests, evergreen forests, and wetland/riparian. We classified cropland sites into four sub-categories: annual crops, vegetable crops, orchards, and vineyards. Lastly, land cover details, e.g., specific crop type or primary species of native vegetation, were identified from site PI comments, imagery, and literature review. An example of these station land cover and land use information is shown in Table 1, and they are included in the metadata table with this dataset.

#### 2.2.8 Key similarities and differences with the FLUXNET2015/ONEFlux approach

Many EC stations within this dataset are also part of recent and past releases of the FLUXNET2015 dataset due to their open data policies and high data quality [1,5]. The main reason that some stations are part of the FLUXNET2015 dataset and are not part of this dataset is that we required each station to include measurements of all four major energy balance variables, and some flux sites in the FLUXNET2015 are missing one or more, typically measurements of soil heat flux. This dataset also includes flux stations that have not yet been shared or incorporated into a FLUXNET2015 release. This dataset also includes a few Bowen Ratio and lysimeter stations as opposed to being limited to EC systems. The largest similarity between FLUXNET2015 and this dataset is that we followed the same approach for energy balance closure correction which is set forth by the ONEFlux daily data processing pipeline, although we did introduce slight variations described in Section 2.2.4 and in Volk et al. [1].

The main difference in our post-processing steps and those implemented by ONEFlux is the method of gap-filling of half-hourly fluxes. We applied a more conservative and simpler approach (linear interpolation) that includes a limit on how many sub-daily gaps could be filled over daytime and nighttime windows (see Section 2.2.2). The ONEFlux process uses the Marginal Distribution Sampling gap-filling method for heat flux variables [5,18] which may utilize longer gap-filling windows and often results in less gaps in the daily averaged fluxes as compared to our method which is more conservative. In addition, this dataset did not apply gap-filling to variables other than LE, H, Rn, G, air temperature, shortwave radiation, and wind speed whereas ONEFlux applies gap-filling to all meteorological variables when possible. Other differences include additional metrics on energy balance closure such as an estimation of random data uncertainty, and several other variables such as carbon dioxide flux and ecosystem productivity metrics which are incorporated in FLUXNET2015 but not included in this dataset [5]. There are also several meteorological variables in this dataset that are not in FLUXNET2015, e.g., potential clear sky radiation and reference ET. Because this dataset is focused on ET, we also applied an air temperature correction to the latent heat of vaporization before computing ET from LE, and applied gap-filling to daily ET using gridded climate data; this does not apply to the FLUXNET2015 dataset as it does not include precomputed ET values.

## Ethics Statements

This work does not involve studies of humans or animals.

## Disclaimer

Trade names are necessary to report factually on available data; however, the USDA neither guarantees nor warrants the standard of the product or service. The use of the name by USDA implies no approval of the product or service to exclude others that may also be suitable. Any use of trade, firm, or product names is for descriptive purposes only and does not imply endorsement by the U.S. Government.

## CRediT authorship contribution statement

**J.M. Volk:** Conceptualization, Methodology, Formal analysis, Software, Validation, Writing – original draft, Writing – review & editing, Data curation, Visualization. **J.L. Huntington:** Project administration, Supervision, Funding acquisition, Conceptualization, Methodology. **F. Melton:** Project administration, Funding acquisition, Writing – review & editing, Conceptualization, Methodology, Data curation, Investigation. **B. Minor:** Data curation, Writing – original draft. **T. Wang:** Data curation, Investigation. **S. Anapalli:** Data curation, Investigation, Writing – review & editing. **R.G. Anderson:** Data curation, Investigation. **S. Evett:** Data curation, Investigation. **A. French:** Data curation, Investigation. **R. Jasoni:** Data curation, Investigation, Writing – original draft. **N. Bambach:** Data curation, Investigation. **W.P. Kustas:** Data curation, Investigation, Writing – original draft. **J. Alfieri:** Data curation, Investigation. **J. Prueger:** Data curation, Investigation. **L. Hipps:** Data curation, Investigation. **L. McKee:** Data curation, Investigation. **S.J. Castro:** Data curation, Investigation. **M.M. Alsina:** Data curation, Investigation. **A.J. McElrone:** Data curation, Investigation. **M. Reba:** Data curation, Investigation. **B. Runkle:** Data curation, Investigation. **M. Saber:** Data curation, Investigation. **C. Sanchez:** Data curation, Investigation. **E. Tajfar:** Data curation, Investigation. **R. Allen:** Methodology, Conceptualization. **M. Anderson:** Methodology, Conceptualization, Writing – review & editing.

## Declaration of Competing Interests

The authors declare that they have no known competing financial interests or personal relationships that could have appeared to influence the work reported in this paper.

## Data Availability

Post-processed data and graphical tools for a CONUS-wide eddy flux evapotranspiration dataset (Reference data) (Zenodo).
